# Hair care product use among pregnant women of color: protocol for a feasibility educational intervention

**DOI:** 10.3389/frph.2025.1694088

**Published:** 2026-01-22

**Authors:** Felice Tsui, Chrystelle L. Vilfranc, Adana A. M. Llanos, Lauren C. Houghton, Jamirra Franklin, Vaishnavi More, Katherine E. Manz, Kurt D. Pennell, Mary Beth Terry, Peggy Shepard, Emily Barrett, Desiree A. H. Walker, Jasmine A. McDonald

**Affiliations:** 1 Department of Epidemiology, Mailman School of Public Health, Columbia University Irving Medical Center, New York, NY, United States; 2 Herbert Irving Comprehensive Cancer Center, Columbia University Irving Medical Center, New York, NY, United States; 3 School of Engineering, Brown University, Providence, RI, United States; 4 WE ACT for Environmental Justice, New York, NY, United States; 5 Department of Biostatistics and Epidemiology, Rutgers School of Public Health, Piscataway, NJ, United States; 6 Young Survival Coalition, New York, NY, United States

**Keywords:** endocrine disruptors, maternal exposures, hair care products, educational intervention, pregnancy, population health, environmental health literacy

## Abstract

**Background:**

Endocrine-disrupting chemicals (EDCs) disrupt hormonal regulation and pose health risks. Phthalates, common in personal care products, contribute to disparate chemical exposures among different demographic groups, notably impacting critical life stages like pregnancy and postpartum.

**Objective:**

Using an environmental health literacy framework, we designed an educational intervention for pregnant Women of Color to highlight the health risks of phthalates in hair care products. The intervention aimed to measure behavioral changes toward phthalate-free products through self-reporting and urinary phthalate metabolite levels and explore factors influencing hair care practices during pregnancy.

**Methods:**

In collaboration with multidisciplinary academicians, environmental health, and breast cancer advocates, we developed a virtual educational intervention during the COVID-19 pandemic. Components included a facilitated presentation, an educational video, and a semi-structured interview guide that was refined through feedback. Data collection involved baseline and follow-up sessions, sociodemographic data, hair product usage, behavior related to phthalate-containing products, and urine sample collection. To provide proof of methodological principle, we examined individual change over time from questionnaire data and targeted exposomics analysis of urinary phthalate compounds among women with baseline and follow-up data.

**Results:**

Educational materials were developed in English and Spanish. Enrollment occurred from March 2021 to June 2022, involving participants in the second or third trimester of pregnancy. Women enrolled before 31 weeks gestation, completed a baseline assessment and at least one follow-up assessment, while those at ≥31 weeks gestation completed a baseline assessment and one postpartum follow-up assessment. Forty-six participants enrolled, with 31 completing the intervention, and 42 urine samples collected. Women who completed the educational intervention were slightly older than those women who did not attend an intervention session [mean age (SD) 31.0 (5.8) vs. 27.5 (5.4)], respectively. Product and brand use decreased over time, and portions of participants exhibited reductions in six different low molecular weight phthalate metabolites (27%–73% reductions).

**Significance:**

This intervention was shaped by a collaborative effort that ensured its cultural relevance, linguistic inclusivity, and alignment with community needs, amplifying its potential impact on reducing the unequal burden of environmental exposures in marginalized communities.

**Clinical Trial Registration:**

NCT04493892.

## Introduction

Endocrine-disrupting chemicals (EDCs) are a widespread group of contaminants that can interfere with the body's natural hormonal regulation (1). They have properties that can promote cancer, such as increasing cell growth and influencing epigenetic changes (2–4). While EDCs are present in our environment, exposure to them is not uniform. They are commonly found in personal care products, and the use of these products varies by gender, race, and ethnicity, leading to unequal exposure and unequal health outcomes (5, 6). For instance, femme-identifying individuals tend to use more personal care products than those identifying as masculine (7), resulting in higher EDC exposure (8, 9). Furthermore, on average, Black women use more hair products while Asian American Pacific Islander women used fewer hair products than Non-Hispanic White women (7), and Hispanic and Asian American Pacific Islander women use more cosmetics than Non-Hispanic White and Non-Hispanic Black women (10).

Hair care products often contain EDCs like phthalates for fragrance and hair flexibility, parabens as preservatives, and estrogens for stimulating hair growth (11–13), but the range of hair care product categories is extensive due to diverse hair textures. In particular, Black women are frequent and long-term users of hair care products, including chemical straighteners, hair oils, and leave-in conditioners, with usage often starting at a young age, creating racial and ethnic disparities in exposure from early life (14–16).

Given that EDC exposures occur throughout one's life, it is appropriate to apply a life course epidemiological framework to reduce EDC exposures that in turn reduce risk of chronic diseases (17). The pregnancy/postpartum window is a crucial period for EDC exposure mitigation. Animal studies provide evidence that prenatal EDC exposures can negatively affect offspring's health, including potentially contributing to hormone imbalances that may influence pubertal development and breast cancer risk (18, 19). High phthalate metabolite concentrations in pregnant women have been associated with offspring obesity and neurological problems (19), preterm birth (20–22), and long-term hormone-related issues, including early puberty, uterine fibroids, endometriosis, polycystic ovarian syndrome, and breast cancer (5, 23–29). The overwhelming evidence on EDC exposure and adverse health outcomes, combined with the disproportionate exposure burden on socially disadvantaged groups, underscores the importance of educational interventions for pregnant women during this critical developmental period. Although epidemiological data is limited, studies have shown that personal care product use among pregnant women is associated with higher levels of urinary phthalates, with concentrations varying based on the type of personal care product used (30–33). Additionally, studies suggest that urinary phthalate metabolite concentrations differ by social demographics and racial and ethnic groups (33), with pregnant racial and ethnic minorities, including a New York City cohort of women (34), showing higher concentrations (35, 36).

The Let's Reclaim Our Ancestral Roots (ROAR) Pilot Study was designed for the diverse population of Northern Manhattan in New York City. This protocol outlines the methodology used in the pilot study, which aimed to (1) offer pregnant Women of Color information on the ways in which certain chemicals in hair care products might contribute to health disparities, (2) assess the study's influence on the expecting mother by measuring changes in product/brand use, and (3) test for potential exposure to phthalates over the study period. The pilot study focused on reducing phthalate exposure based on previous intervention findings of adverse health effects (37) and the widespread presence of phthalates, which have been detected in human breast milk, urine, amniotic fluid, and the placenta (38–48).

## Methods

### Development of study team to address community needs

A multidisciplinary team of academicians and health advocates representing diversity in gender, race, ethnicity, age, and socioeconomic positions developed the Let's ROAR Pilot Study. The multi-dimensional collaborative included molecular and life course epidemiologists with expertise in chemical exposures and health disparities, environmental exposure scientists, environmental health and breast cancer advocates, implementation science/mixed methodologists, and multi-lingual experts in community outreach and engagement whose perspectives converged for the collective purpose of environmental justice, health equity, and prioritization of the community. This team was built upon and galvanized by the belief that to address community needs, interventions must be designed and developed not only for the community, but with the community.

The conceptualization and execution of the study was an intensely collaborative process that took place on virtual Zoom meetings and email due to the COVID-19 pandemic. This spanned the development and submission for pilot funding, the selection of frameworks and principles, the design of the study, and the development and distribution of materials to participants. We jointly hypothesized that an educational intervention targeting pregnant Women of Color, focusing on EDC exposure, hair care products, and their implications for fetal and early childhood health, as well as maternal health outcomes, would lead to changes in hair care product usage. We based this hypothesis on the idea that pregnant women may be motivated to modify their behaviors during pregnancy for the well-being of their infants (49). We also drew on previous behavioral interventions (47, 50–55) designed to address environmental health concerns during critical periods of exposure susceptibility (37, 56–61) (Supplementary Table S1).

### Theoretical frameworks and behavioral models

For our study approach, we adopted Finn and O'Fallon's Environmental Health Literacy framework. This framework suggests that providing individuals with information about their environmental exposures empowers them to make health-conscious decisions regarding their interactions with the environment (9). To guide our pilot study development, we adapted Bloom's Taxonomy for Educational Objectives to the Let's ROAR objectives, as detailed in Supplementary Figure S1.

**Figure 1:**
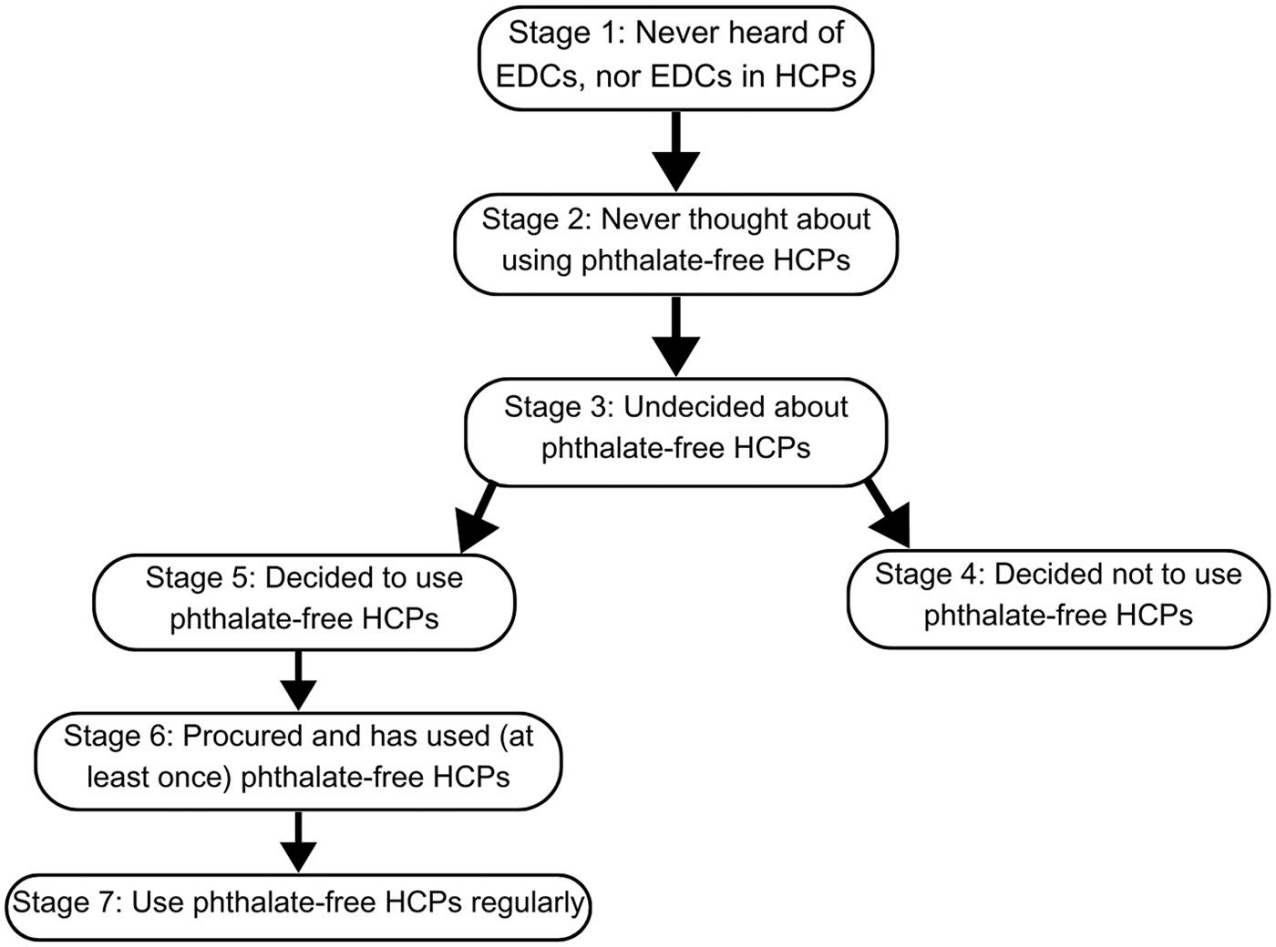
Precaution adoption process model (PAPM) stages. We present the seven stages that constitute the precaution adoption process model (PAPM) that was applied in the development of questionnaires to assess baseline awareness and behavioral action around phthalate-containing hair care products. We asked Stage 1 through Stage 7 related questions at baseline. At post-intervention, we asked Stage 2 through Stage 7, as after the educational intervention, one cannot accurately report never hearing about phthalate-containing hair care products.

In creating the educational intervention, which included a presentation and embedded video, we utilized Marshall Ganz's Public Narrative: Self, Us, Now framework. This framework centers on the learning process of storytelling, listening, and reflecting (62). To assess behavioral changes, we applied the Precaution Adoption Process Model, a theoretical model consisting of seven stages that explains how individuals progress from awareness of a health behavior to making decisions and translating those decisions into action, as depicted in Figure 1 (63).

Our semi-structured focus group drew from the model of discrete choice (64) and sought feedback on the factors that Women of Color consider when making decisions about hair care products. Drawing from consumer theory, which posits that rational consumers assess various options, assign values or preferences to each option, and then choose their preferred one (64), we aimed to understand what factors Women of Color consider when selecting hair care products. Interestingly, a study involving 68 pregnant French women found that ingredients were seldom a primary criterion in their product choices (65). Therefore, we wanted to explore the criteria that Women of Color prioritize when purchasing hair care products.

### Non-randomized clinical trial study approach

The development of this clinical trial, from the study design to the protocols, was an iterative process involving extensive feedback from the study team and key informants.

#### Development of the virtual educational intervention materials

Initially, we applied the Self, Us, Now framework (62, 66) to structure the educational intervention presentation and video. This framework comprised five parts: Introduction, Background, Educational Video Viewing, Reflection and Reiteration, and Discussion. Each part had specific objectives, such as personalizing hair care product use, addressing chemical exposure inequities among Women of Color, and fostering open dialogue.

The educational intervention video, lasting seven minutes, focused on defining EDCs, critical windows of susceptibility to environmental exposures, examples of phthalate exposure and associated health effects, the benefits of limiting exposure to phthalate-containing hair care products, and practical tips for reducing such exposure. After the educational intervention session, participants received a post-session handbook via email, which summarized session topics, provided tools for checking product ingredients, and suggested alternative, less-toxic hair care product brands for the mother and baby. Criteria to select products were most compliant with the restrictive ingredients list used by the clean beauty retailer Follain, now owned by Credo Beauty. At the time, Follain had one of the most scientifically backed restrictive ingredient lists.

#### Development of the semi-structured interview guide

Two versions of the semi-structured interview guide were created. Initially, one version was offered during a separate 1-hour focus group session following the educational intervention session. To reduce participant burden, we later developed a modified version, which was integrated into the closing discussion session of the educational intervention. Both versions explored participants' reflections on past and present hair care practices from childhood to pregnancy and the attributes they considered when choosing hair care products. We aimed to identify decision-making factors beyond just ingredients, considering that participants might not solely base their decisions on ingredients alone.

### Data collection

Our secondary objective was to quantify behavioral changes in the use of phthalate-free hair care products. We hypothesized that the educational intervention would increase awareness of phthalate chemicals in hair care products among pregnant Women of Color and promote progression across stages of the Precaution Adoption Process Model. We adapted existing data collection materials and developed new ones as needed. All questionnaires were self-administered, available online, and offered in both English and Spanish. These included:

- Sociodemographic Questionnaire: Gathered data on participants' sociodemographic information, overall health, and pregnancy-related factors.
- Hair Care Product Use Questionnaire: Adapted from prior works to recall hair care product usage within the last 24–48 h (7).
- Precaution Adoption Process Model Questionnaire: Assessed baseline awareness and behavioral actions regarding phthalate-containing hair care products (67).
- Follow-up Questionnaires: Assessed behavioral changes post-educational intervention.

**Figure 2:**
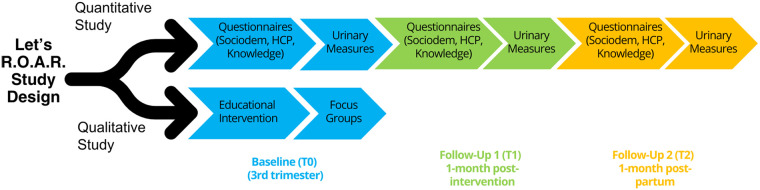
Let's reclaim Our ancestral roots (R.O.A.R) Study Design. We outline the qualitative and quantitative components of the mixed methods study design that aimed to deliver an educational intervention to reduce phthalate metabolite exposures and to quantitate behavioral change in the use of phthalate-free hair care products in pregnant Women of Color.

We required participants to complete a baseline assessment, with two follow-up assessments: one around the 34th gestational week and the other approximately 1 month postpartum. Women enrolling at 31 or more gestational weeks completed a baseline assessment and one follow-up assessment at 1 month postpartum (Figure 2).

### Study recruitment & enrollment protocol

In collaboration with the Columbia University Irving Medical Center Department of Obstetrics and Gynecology and using targeted Instagram advertisements (Meta Platforms), our study aimed to recruit 50 Women of Color who were in their 27–30 weeks of pregnancy. These participants were required to be English and/or Spanish speakers and residents of New York City boroughs. We maintained contact and provided follow-up through phone calls, text messaging, and email. Baseline enrollment commenced in March 2021 through Instagram and in October 2021 with the Department of Obstetrics and Gynecology.

Upon confirming eligibility, participants underwent the consent process and were enrolled in the study. Enrolled participants received a welcome package that included tea for promoting the health of pregnant women and Let's ROAR study-branded stickers, both made by Woman of Color-owned businesses.

Subsequently, we scheduled all participants for a one-hour virtual educational intervention session. The educational intervention sessions were conducted via Zoom, available in both English and Spanish. After completing the educational intervention session, we invited English-speaking participants to attend a separate one-hour semi-structured focus group session to collect qualitative data. Initially, three one-hour semi-structured focus groups were conducted in English via Zoom as standalone sessions. Later, protocol changes were implemented so that the remaining four focus group sessions were integrated into the educational intervention sessions and were offered in both English and Spanish. Participants had the option to control their video and change their displayed name for anonymity within the Zoom platform. All sessions were recorded and transcribed. While Instagram advertisements were exclusively in English, all other study materials were culturally tailored and translated into Spanish. See Vilfranc et al. for further details (68).

### Biospecimen collection

We asked all participants to provide baseline urine samples before the educational intervention session and subsequent samples at each follow-up assessment. Study staff mailed urine collection kits to participants with instructions and prepaid return packaging. Laboratory staff processed, aliquoted, and stored urine samples within 48 hours of collection. We collected 42 urine samples from 21 study participants, which included 5 participants with a baseline sample only, 11 participants with baseline and 1 follow-up sample and 5 participants with samples from all 3 visits.

#### Biospecimen preparation

In brief, urine samples were sent to the Environmental Exposure Lab at Brown University. Samples were assessed for specific gravity to adjust for urine dilution using a refractometer. Samples were extracted based on EPA Method 8061A for phthalates and phthalate esters and the pH of each sample was measured prior to extraction and adjusted to pH 5.0 using phosphoric acid (69). The Supplemental Materials detail the biospecimen preparation protocol for the following 17 compounds individually purchased from Accustandard: dimethyl phthalate (DMP), diethyl phthalate (DEP), dibutyl phthalate (DBP), di-n-octyl phthalate (DNOP), Bis(2-ethylhexyl) adipate (DEHA), Bis(2-ethylhexyl) phthalate (DEHP), benzyl butyl phthalate (BBP), monobutyl phthalate (MBP), monobenzyl phthalate (MBZP), mono-isobutyl phthalate (MIBP), monomethyl phthalate (MMP), monoethyl phthalate (MEP), mono(2-ethyl-5-oxohexyl) phthalate (MEOHP), mono(2-ethyl-5-hydroxyhexyl) phthalate (MEHHP), mono(2-ethylhexyl) phthalate (MEHP), monoisononyl phthalate (MINP), mono(3-carboxypropyl) phthalate (MCPP), monocyclohexyl phthalate (MCHP), and mono-n-octyl phthalate (MOP).

#### GC-HRMS for targeted analyses of phthalate metabolites

In brief, sample extracts were analyzed using a Thermo GC-Orbitrap Q Exactive mass spectrometer equipped with a Thermo Trace 1300 GC and TriPlus RSH autosampler. Targeted analysis was performed using Thermo TraceFinder software (EFS Version 4.1 SP1) and the extracted ion chromatogram (XIC) was used for quantification using the most abundant peak in the mass spectrum for each phthalate. The Supplemental Materials details sample extraction and the targeted analysis protocol for parent phthalate and phthalate metabolites. We do not present data from the non-targeted exposomics analysis. For Quality Assurance/Quality Control (QA/QC), please see Supplementary Table S2 for quality control blanks and Supplementary Table S3 for matrix spikes and recovery data.

### Statistical analysis

We conducted *t*-tests and chi square to examine differences between participants who attended an educational intervention session compared to participants who did not (Table 1) and between participants who provided a urine sample compared to those who did not (Supplementary Table S4). We conducted descriptive analyses for self-report and targeted urinary phthalate data at baseline and follow-up 1 (hereafter referred to as follow-up). We did not conduct descriptive analyses at follow-up 2 given the sample size (*n* = 5). For women with paired baseline and follow-up data, we calculated the mean (SD) for the total number of products used (*N* = 13) and brands used (*N* = 11) within 24–48 hours. Urinary concentrations of each chemical compound had to be detected in at least 70% of samples to be included in analyses; MMP (100% non-detect), MCHP (95% non-detect), and DNOP (85% non-detect) were excluded. All samples were analyzed as continuous variables, including those below the limit of detection (LOD). Concentrations below the LOD without an output concentration were replaced with LOD√2. We log-transformed the data to account for non-normal distribution. Supplementary Table S5 provides the low molecular weight (LMW) parent phthalate and associated metabolites corresponding LODs. LMW phthalates were the primary target of the intervention given the high prevalence in hair care [parent/(metabolite)]: DBP/(MBP), DEP/(MEP), DMP, (MIBP) (Supplementary Table S5) (86). We calculated the geometric mean and standard deviation, percentiles (25th, 50th, and 75th), and minimum and maximum in ng/mL for LMW phthalate compounds at baseline (*N* = 20) and follow-up (*N* = 17) (Supplementary Table S6). We then restricted analyses to women who attended the virtual intervention with baseline and follow-up data and calculated the percent change in exposure (*N* = 16): [(follow-up (ng/mL) – baseline (ng/mL))/(baseline (ng/mL))]*100%. Lastly, to provide context to quantitative changes in the LMW phthalate metabolites, we integrate qualitative statements from two focus group participants using joint display (Figure 4).

**Table 1:** Sociodemographic data of enrolled participants by completion of the educational intervention.

Sociodemographic data	Educational intervention (*N* = 31)	Non-educational intervention (*N* = 15)	*P*-value
Age, year	31.03 ± 5.80 (22–41)	27.47 ± 5.42 (19–37)	0.053
Race, *n* (%)			
Black Hispanic	9 (29.0)	4 (26.7)	0.97
Black non-Hispanic	6 (19.4)	2 (13.3)	
AI/AN Hispanic	1 (3.2)	0	
White Hispanic	2 (6.5)	1 (6.7)	
Greek Hispanic	1 (3.2)	0	
Taino Hispanic	1 (3.2)	0	
Asian non-Hispanic	1 (3.2)	0	
Black and AI/AN	1 (3.2)	0	
Other Hispanic	7 (22.6)	7 (46.7)	
Refuse to report and Hispanic	2 (6.5)	1 (6.7)	
Country, *n* (%)			
Dominican Republic	12 (38.7)	8 (53.3)	0.95
United States	15 (48.4)	7 (46.7)	
Guinea	1 (3.2)	0	
Togo	1 (3.2)	0	
Liberia	1 (3.2)	0	
St. Thomas	1 (3.2)	0	
Language, *n* (%)			
English	29 (93.5)	12 (80.0)	0.31
Spanish	2 (6.5)	3 (20.0)	
Pregnancy, week	31.35 ± 3.55 (22–37)	31.40 ± 2.82 (26–36)	0.96

The Internal Review Board at Columbia University Irving Medical Center approved the study.

## Results

**Figure 3:**
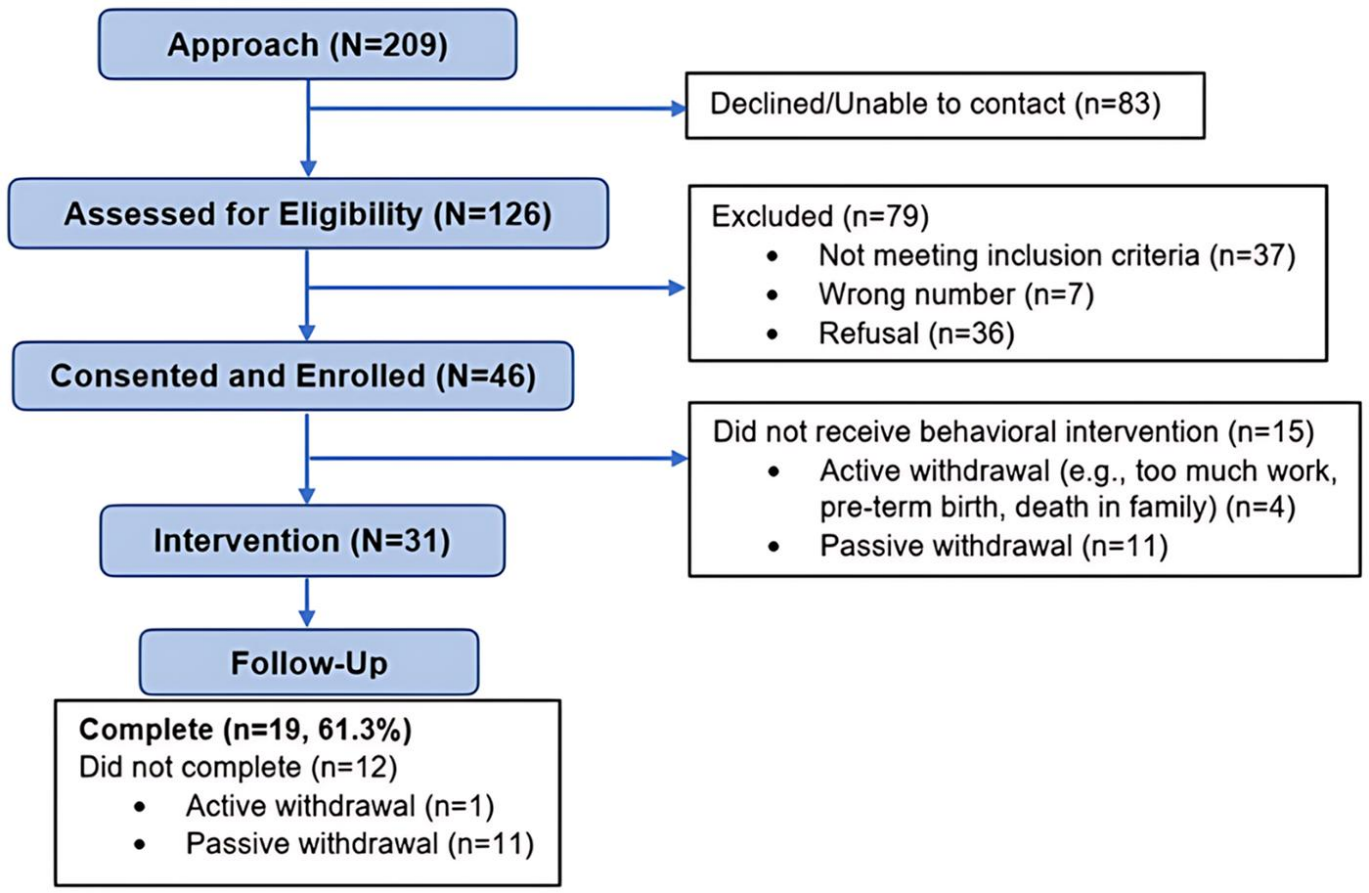
Consort diagram. Consort diagram depicts the cadence of study participants through assessment for eligibility, consent and enrollment, receipt of intervention, and follow-up.

Figure 3 details the consort diagram for this feasibility pilot study. Among the 209 women recruited, 39.7% of women declined or were unable to be contacted for screening. Among the 126 women screened for eligibility, 62.7% were excluded for not meeting inclusion criteria (*n* = 37), refusing study consent and enrollment (*n* = 36), or deemed eligible but unable to be contacted for consent and enrollment. Of the targeted number of 50 enrolled participants, we consented and enrolled 46 women with 67.4% (*n* = 31) receiving the behavioral intervention; the other 15 withdrew before receiving the intervention.

The overall cohort of 46 women were on average 31.37 (SD 3.30) weeks pregnant and racially and ethnically diverse. Those who attended an intervention session (*n* = 31) were not significantly different than those who did not attend an intervention session, with one exception. Women who completed the educational intervention were slightly older than those women who did not attend an intervention session [mean age (SD) 31.0 (5.8) vs. 27.5 (5.4), respectively] (Table 1). Similarly, women who provided a urine sample were slightly older than those that did not (Supplementary Table S4).

Vilfranc et al. details the study's thematic coding of qualitative data in Dedoose that identified two influential life stages and three intersecting themes (i.e., products, individuals/entities of influence, and alignment with inner and outer beauty) that impacted participants' agency in hair care practices and product choices (68).

**Figure 4:**
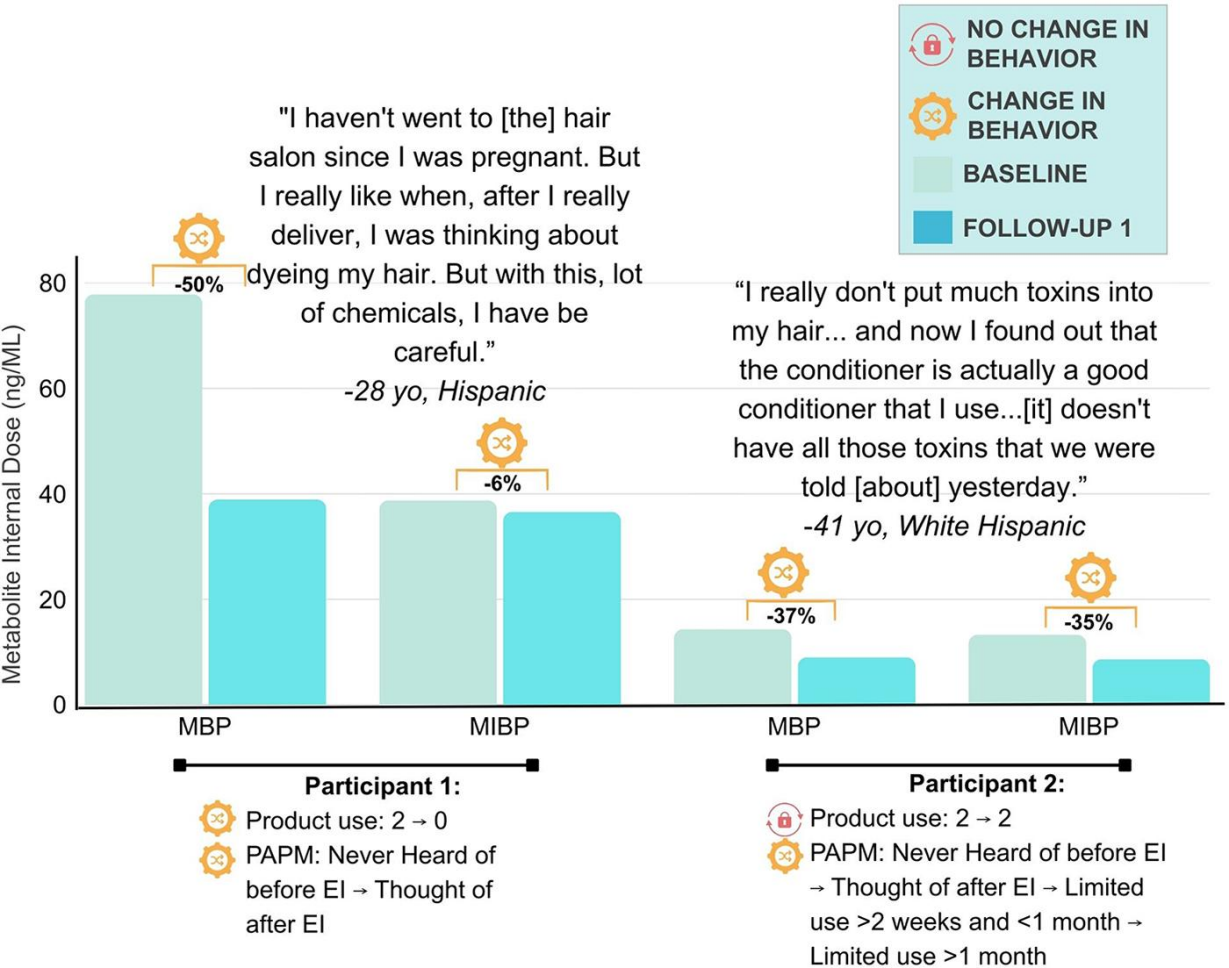
MBP and MIBP phthalate metabolite concentrations over time. We illustrate a mixed-method narrative for 2 participants. The *X*-axis includes urinary phthalate metabolite data for MBP and MIBP at baseline and follow-up 1. The *Y*-axis is the metabolite's internal dose (ng/ml). We present the total number of self-reported hair products that were used in the last 24–48 h at baseline and at follow-up 1 assessments. We present available self-reported data on progression across the Precaution Adoption Process Model (PAPM; pre-intervention, post-intervention, follow-up 1, follow-up 2). Within the figure, PAPM “never heard of” refers to never hearing about phthalates (Stage 1); “thought of” refers to participant having decided they want to reduce/eliminate the use of hair care products containing phthalates (Stage 5); “limited use” refers to participants reporting limited or the elimination of use of hair care products containing phthalates for more than 2 weeks but less than 1 month *or* for more than 1 month (Stage 7). The arrows represent the progression across visits. We present a quote from the post-intervention focus groups for each participant. The orange gear indicates a change in behavior, an observed decline in exposure. The red lock indicates no change in behavior, no change in exposure observed. EI, Educational Intervention.

From baseline to follow-up, the average number of products used was mean (SD) 2.3 (1.4) to 1.9 (1.4), and the number of brands used was mean (SD) 2.0 (1.7) to 1.2 (0.7). The proportion of participants exhibiting reductions in LMW phthalate compounds included: 73% DBP, 60% MBP, 47% MIBP, 33% DEP, 33% DMP, and 27% MEP. Figure 4 provides a joint display of change in behavior and MBP and MIBP in 2 women with corresponding quotes. MEP results were varied: participant 1 had a 100% reduction, with follow-up levels below the LOD; and, participant 2 had baseline values below the LOD, but had a concentration of 37.02 ng/ml at follow-up. Our mixed-methods narrative for participant 1 and 2 objectively demonstrated a decrease in MIBP and MBP exposure; however, self-reported measures differed. Participant 1 self-reported transitioning from PAPM Stage 1 (never heard of phthalates) to Stage 5 (deciding to act to reduce phthalate exposure) and reports a reduction in product use from 2 to 0. In contrast, participant 2 self-reported transitioning from PAPM Stage 1 to Stage 7 (maintaining reduction of phthalate exposure) and reports no reduction in product use. While participant 2 suggested that their behavior would unlikely change, they showed consistent self-reported progression through the PAPM with evident change in metabolite concentrations. Quotes from the participants suggest that the intervention educated and encouraged greater consideration of the ingredients present in the hair care products of both women.

## Discussion

To our knowledge, this marks the first educational intervention aimed at reducing the use of phthalate-containing hair care products among pregnant Women of Color. The Let's ROAR study was designed to raise social and cultural awareness of what EDCs are, the health impacts of EDC exposure during critical windows of development, and how to limit exposure to these substances in the context of personal hair care, with a delivery that encouraged and embraced the impact that culture and normative beliefs can have on behavior. This protocol paper outlines the frameworks and methodology used in developing a virtual educational intervention within a key window of susceptibility in order to reduce the use of phthalate-containing hair care products to ultimately impact downstream chronic conditions for mother and child. The mixed-methods design facilitated the collection of quantitative and qualitative data that will be used to inform the testing of a larger-scale intervention.

Health interventions have been an effective risk-reduction strategy to guide consumer choices away from environmental exposures of concern such as EDCs (47, 50, 70). However, prior studies are limited by a lack of psychosocial evaluation and/or the lack of cohort diversity. Here, we designed an intervention informed by Finn and O'Fallon's Environmental Health Literacy framework which suggests that providing people with knowledge about their environmental exposures will empower them to make health-protective decisions on how they engage with their environment (9). It allows for greater understanding of specific risks, reductions in exposures, and improvement of health outcomes for individuals and communities, while enhancing coalition building efforts with the goal of achieving environmental justice.

Women are the largest consumers of PCPs, with use varying by sociodemographic characteristics such as race and ethnicity (14–16). Women of African and/or Latin/Hispanic descent are among the most frequent and prolific consumers of certain hair care products, such as chemically ladened hair straightening products and hormone-containing hair care products, which are more commonly used because of a Eurocentric view of beauty that idealizes straight hair (68, 71, 72). There are limited studies focused on the prevalence of PCP use, including hair care products, among pregnant women. However, the existing literature suggests that PCP use in pregnant women is correlated with higher concentrations of urinary phthalates, that urinary metabolite concentrations vary by PCP type, and that urinary metabolite concentrations differ by sociodemographic factors including race and ethnicity (30, 31, 33–36). For example, a study examining PCP use among pregnant women and new mothers in New York City during the COVID-19 pandemic (July 2020–June 2021) found that non-Hispanic Black women used approximately two more product types compared to non-Hispanic White women (73).

Pregnancy is a critical window where exogenous exposure to harmful chemicals has been associated with adverse health effects for the child and mother (74). For example, the ERGO (Environmental Reproductive and Glucose Outcomes) study on 154 pregnant women in Boston, Massachusetts found that non-Hispanic Black women who reported daily use of hair oils during pregnancy delivered significantly earlier (by 8.3 days) compared to non-hair oil users. Phthalate-specific studies have found associations with health effects from *in utero* exposure including elevated BMI, metabolic abnormalities, respiratory issues and asthma, and neurodevelopmental disorders (75–79). The adverse health outcomes in mothers are also numerous, including reduced fecundity/fertility, pregnancy complications, adverse later-life cardiometabolic health outcomes, impaired glucose tolerance (i.e., sensitivity to sugar, a risk factor in diabetes), and more (19, 80–82). Moreover, given the elevated risk of breast cancer lasting 10 years or more postpartum, exposures in the postpartum period are also of concern (83). This suggests the perinatal and postpartum periods as critical stage that influence fetal development and maternal outcomes (84). Thus, the perinatal and postpartum periods are perfect windows to intervene on.

Given the small numbers of women with completed matched baseline and follow-up data, the results we presented as proof of methodological principle may be due to chance. However, the convergence of qualitative and quantitative data adds rigor. Following CDC and NHANES guidelines (69), we chose to highlight urinary LMW phthalate metabolite concentrations as our objective measure for changes in behavior change. The proportion of women with MEP reductions were much lower than MBP and MIBP. However, MEP was detected in only 35% of samples, which is unexpectedly low, given that >90% of women usually show measurable levels, depending on factors such as population and measurement method. Therefore, the low detection of MEP in our pilot study demonstrates that the measurement should be viewed with caution and contextualized with other phthalate metabolite concentrations. We provided mixed-method narratives on 2 participants confirming that the reduction in exposures aligned with participants' lived experience of reducing exposure.

In closing, given that it is common for women to alter their routines to reduce certain exposures that may be harmful to the baby, such as avoiding deli meats, fish, and more. An intervention tailored to pregnancy, where the mother is primed to make health changes (49), has potential for intergenerational health impacts not only *in utero* but also postpartum. Environmental exposure intervention studies to date have been integral in solidifying the importance of behavioral change in the mitigation of associated health outcomes, specifically in childhood, puberty, and motherhood (37, 47, 50–61). However, few have considered the intersection of race, ethnicity, gender, and critical windows of exposure in the use of PCPs. The HERMOSA study is an exception, focusing on the impacts of chemicals found in PCPs used during the critical window of puberty in 15 Latina girls (85). Likewise, the PREVED (Pregnancy, PREVention, and Endocrine Disruptors) study developed an intervention to educate 210 pregnant women of unspecified sociodemographic makeup on how to identify and choose products to mitigate domestic air pollution, food pollutants and PCPs (56). Like the PREVED study that evaluated psychosocial changes including self-esteem, perceived health, risk perception, and level of concern for EDCs, Let's ROAR explored similar psychosocial changes in addition to the individual's hair journey from childhood to pregnancy. We are unaware of any educational behavioral intervention specifically addressing hair care product use during the perinatal window of exposure in WOC, thereby providing the impetus for this feasibility study. The Let's ROAR pilot study demonstrated self-reported and objective reductions in hair product exposures and in urinary phthalate metabolites. Thus, this study presents a scalable model that empowers vulnerable populations to make informed decisions that mitigate health risks and overall promote positive health outcomes for both individual mothers and their children as well as their collective community.
