# Supplementary material for: Hair care product use among pregnant women of color: protocol for a feasibility educational intervention
Source: Front Reprod Health. 2026 Jan 22;7:1694088. doi: 10.3389/frph.2025.1694088 (PMC12872876; doi:10.3389/frph.2025.1694088)
Supplement: Supplementary file 1 [file Supplementaryfile1.docx]

**Supplementary Materials**

**Hair Care Product Use Among Pregnant Women of Color: Protocol for a Feasibility Educational Intervention**

Supplementary Table 1. Summary Table of Interventions Designed to Address Environmental Health Concerns During Critical Periods of Exposure Susceptibility

Supplementary Figure 1. Adaptation of the Let’s Restore Our Ancestral Roots (Let’s R.O.A.R) Study Objectives from Bloom’s Taxonomy for Educational Objectives.

Phthalates and Phthalate Esters Biospecimen Preparation and *GC-HRMS*

*Biospecimen Preparation and GC-HRMS Protocol*

Supplementary Table 2. Targeted Analysis Data For Blanks

Supplementary Table 3. Targeted Analysis Data For Matrix Spikes And Recovery

Supplementary Table 4. Sociodemographic Data of Participants Who Provided a Urine Sample Compared to Those Who Did Not

Supplementary Table 5. Phthalates and Phthalate Esters Eligible for Analysis Within the Let’s ROAR Pilot Study: Limit of Detection and Percentage of Non-Detect

Supplementary Table 6. Supplemental Table 6. Phthalates and Phthalate Esters Descriptive Statistics

**Supplementary Table 1. Summary Table of Interventions** **Designed to Address Environmental Health Concerns During Critical Periods of Exposure Susceptibility**

|  | **Title** | **Location (Population)** | **Intervention** | **Target Exposure** | **Study Overview** |
| --- | --- | --- | --- | --- | --- |
| 1 | El Ouazzani, H., et al., 2021., Impact of perinatal environmental health education intervention on exposure to endocrine disruptors during pregnancy—PREVED (Pregnancy PreVention, Endocrine Disruptors) study: study protocol for a randomized controlled trial (PMID [35010328](https://pubmed.ncbi.nlm.nih.gov/35010328)) | France (N=268 Prenatal) | Educational (14 months) | Manufactured/industrial food consumption | Open label randomized controlled trial aimed to educate pregnant women about EDC exposure and assess its impact. The primary outcome was the percentage of participants consuming manufactured/industrial food, with secondary outcomes including psychosocial dimensions and EDC concentrations in urine and colostrum. Outcomes were measured over the span of 14 months via questionnaires and urinary samples. |
| 2[ | Gallaway, T.S., et al., 2018., An engaged research study to assess the effect of a 'real-world' dietary intervention on urinary bisphenol A levels in teenagers (PMID 29431133) | United Kingdom (N=94, ages 17-19 years) | Self-administered dietary (7 days) | Bisphenol-A (BPA) levels | Researchers investigated whether dietary changes could reduce BPA exposure. Urinary BPA levels were measured before and after a 7-day intervention. The study found that self-moderating BPA exposure through diet in a real-world setting was challenging to sustain. |
| 3 | Kim, J.H., et al., 2021., Web-based behavioral intervention to reduce exposure to phthalate metabolites, bisphenol A, triclosan, and parabens in mothers with young children: A randomized controlled trial (PMID 34186503) | Online (N=51 mothers with young children) | Web-based behavioral (6 weeks) | P[hthalate](https://www.sciencedirect.com/topics/earth-and-planetary-sciences/phthalate) metabolites, [bisphenol](https://www.sciencedirect.com/topics/earth-and-planetary-sciences/bisphenol) A, [triclosan](https://www.sciencedirect.com/topics/medicine-and-dentistry/triclosan), and [parabens](https://www.sciencedirect.com/topics/medicine-and-dentistry/4-hydroxybenzoic-acid-ester) | A web-based randomized controlled trial was implemented to reduce EDC exposure in mothers with young children. After 6 weeks, the intervention group exhibited significant reductions in urinary phthalate metabolites, bisphenol A, triclosan, and parabens. |
| 4[ | Rudel, R.A. et al., 2011., Food Packaging and Bisphenol A and Bis(2-Ethyhexyl) Phthalate Exposure: Findings from a Dietary Intervention (PMID: 21450549) | California (N=20 participants from 5 families) | Dietary (8 days) | BPA and phthalate metabolites | Researchers measured urinary BPA and phthalate metabolites in families during and after a dietary intervention focused on "fresh foods." Significant reductions in BPA and phthalate metabolite exposures were observed over 8 days when participants' diets were limited to minimally packaged foods. |
| 5 | Yang M, 2014., Effects of Korean red ginseng (Panax Ginseng Meyer) on bisphenol A exposure and gynecologic complaints: single blind, randomized clinical trial of efficacy and safety (PMID: 25063041) | Korea (N=22 females) | Dietary (2 weeks) | Korean Red Ginseng (KRG) | A single-blinded randomized control trial assessed the impact of KRG on BPA exposure and quality of life in females. KRG consumption led to decreased urinary BPA and alleviation of menstrual-associated morbidities and related oxidative stress. |
| 6 | Barrett E.S., et al., 2015, Reducing prenatal phthalate exposure through maternal dietary changes: results from a pilot study (PMID: 25652062) | United States (N=10 low-income pregnant females) | Dietary (3 days) | Phthalate exposure in food processing and packaging | A 3-day dietary intervention pilot study aimed to reduce phthalate exposure; however, phthalate metabolite concentrations did not significantly change during the intervention, suggesting the need for broader measures to lower exposure. |
| 7 | Harley K.G., et al., 2016. Reducing Phthalate, Paraben, and Phenol Exposure from Personal Care Products in Adolescent Girls: Findings from the HERMOSA Intervention Study (PMID: 26947464) | United States (N=100 Latina girls) | Community based participatory research (3 days) | Phthalate metabolites, parabens, triclosan, and BP-3 in personal care products | A youth-led community-based participatory research intervention involving the use of personal care products labeled as free of certain EDCs for 3 days. Results demonstrated that consumers' choices of labeled products could reduce exposure to potential endocrine-disrupting chemicals. |
| 8 | Hagobian T., et al., 2017, Randomized Intervention Trial to Decrease Bisphenol A Urine Concentrations in Women: Pilot Study (PMID: 27726525) | California (N=24 women) | Dietary, cosmetics, and other packaged products (3 weeks) | Reduce BPA exposures from food, cosmetics, and other packaged products. | A randomized control pilot study that involved a 3-week intervention to reduce BPA exposure from food, cosmetics, and packaged products. Women were provided with BPA-free replacement products and daily self-monitored. The intervention successfully decreased urinary BPA concentrations. |
| 9 | Peng C.Y., 2019, Canned food intake and urinary bisphenol a concentrations: a randomized crossover intervention study (PMID: 31352597) | (N=20 college students) | Dietary (1 day) | BPA | A randomized crossover study assigned students fresh or canned foods. Urinary BPA concentrations increased after consuming canned foods but returned to baseline levels within 24 hours, indicating the effectiveness of dietary intervention and a 1-day washout period. |
| 10 | Rutkowska A., et al., 2020, Changes in daily life reduce indoor exposure to selected endocrine disruptors in the home environment: a pilot intervention study (PMID: 32558529) | Poland (N=26 individuals from 9 households) | Indoor home environment (6 months) | Home-related exposure to EDCs | A 6-month pilot study analyzed the impact of lifestyle modifications on exposure to EDCs in households (pre and post urine and dust samples). Participants' lifestyle changes led to significant reductions in home-related EDC exposure and urine concentrations of these chemicals. |
| 11 | Van der Meer T.P., et al., 2021, Endocrine disrupting chemicals during diet-induced weight loss - A post-hoc analysis of the LOWER (Lifestyle, OverWeight, Energy Restriction) study (PMID: 33045228) | Netherlands (N=218 individuals) | Dietary (3 months) | Parabens, bisphenols, and phthalate metabolites | The effect of calorie-restricted weight reduction on urinary EDC exposure in obese individuals. Most phthalates decreased after the 3-month dietary intervention, suggesting that weight reduction can lower EDC exposure. |
| 12 | Harley K.G., et al., 2021, Changes in Latina Women’s Exposure to Cleaning Chemicals Associated with Switching from Conventional to “Green” Household Cleaning Products: The LUCIR (Lifting Up Communities with Interventions and Research) Intervention Study (PMID: 34468180) | California (N=50 Latina women) | Youth-led community-based; Cleaning Products (1 weeks) | 47 chemicals of concern | Latina women participated in personal air monitoring during regular and "green" cleaning product use for 1 week. The switch to green products significantly reduced air concentrations of several carcinogens and endocrine disruptors, highlighting the benefits of choosing eco-friendly cleaning products. |
| 13 | Park S., et al., 2021, Effects of a dietary modification intervention on menstrual pain and urinary BPA levels: a single group clinical trial (PMID: 33563271) | South Korea (N=30 female college students) | Dietary (4-6 weeks, dependent on each participant’s menstrual cycle) | BPA | A 4-6 week dietary intervention study focused on reducing menstrual pain and urinary BPA levels effectively reduced both. |
| 14 | Sessa F, et al., 2021, Effects of a Plastic-Free Lifestyle on Urinary Bisphenol A Levels in School-Aged Children of Southern Italy: A Pilot Study (PMID: 33598445) | Italy (N=130 Children) | Dietary (6 months) | BPA | A pre-test post-test study assessed urinary BPA levels in 130 Italian children over six months, during a diet regimen with reduced plastic food packaging. The results showed the effects of a diet regimen with reduction of plastic food packaging, demonstrating a connection between urinary BPA levels and food packaging. |

**Supplementary Figure 1. Adaptation of the Let’s Restore Our Ancestral Roots (Let’s R.O.A.R) Study Objectives from Bloom’s Taxonomy for Educational Objectives.**


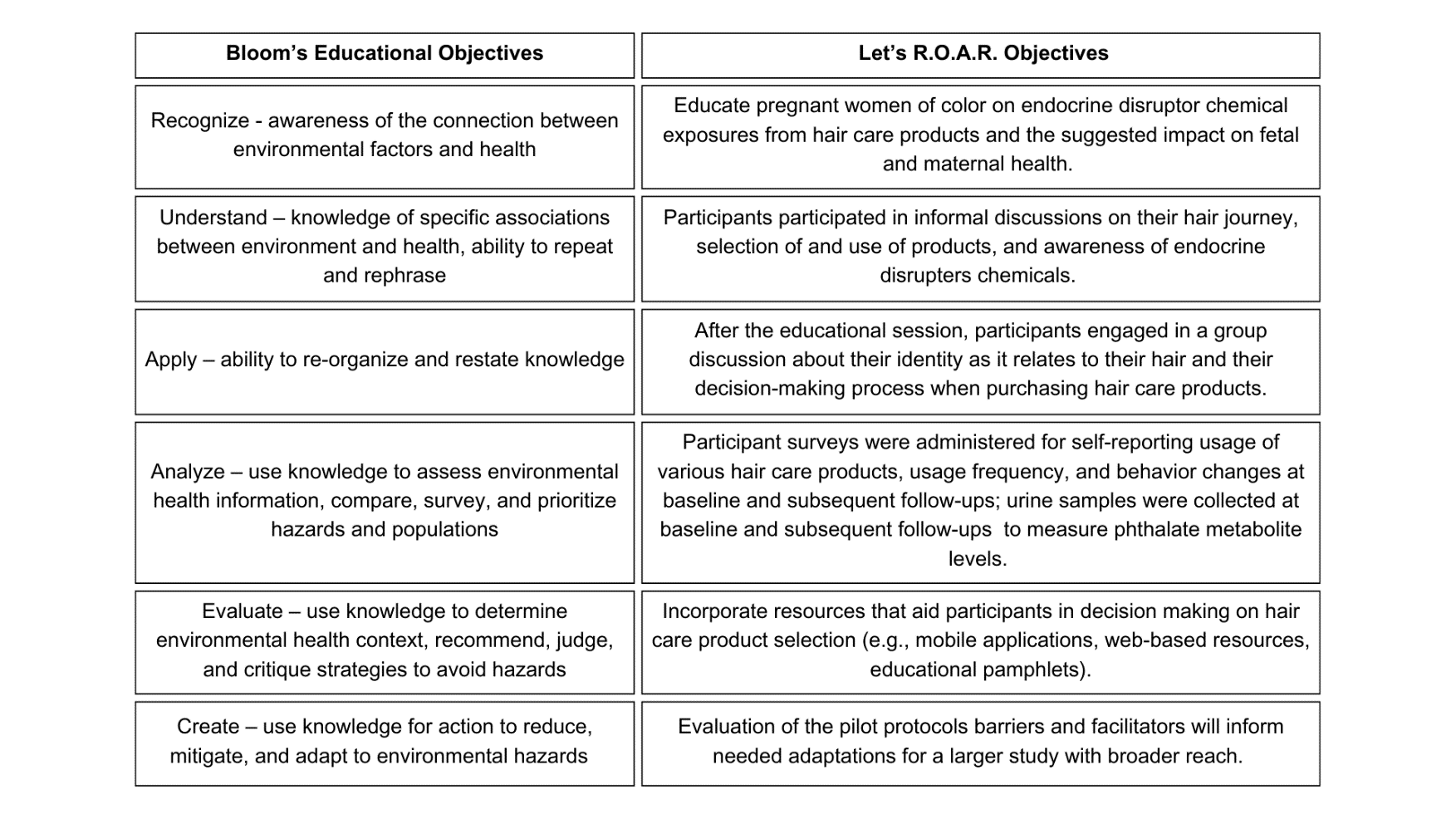


**Phthalates and Phthalate Esters Biospecimen Preparation and GC-HRMS**

***Biospecimen Preparation***

The urine sample were assessed for specific gravity to adjust for urine dilution using a refractometer. Urine samples were extracted based on EPA Method 8061A for phthalates and phthalate esters. Since phthalates are known to hydrolyze at basic pH, the pH of each sample was measured prior to extraction and adjusted to pH 5.0 using phosphoric acid (Silva et al. 2005). A 5 mL aliquot of urine was transferred to an amber glass vial with a cap and spiked with a surrogate standard solution containing diphenyl phthalate, diphenyl isophthalate, and dibenzyl phthalate. The surrogate standards were used to monitor the performance of the extractions and assess extraction recovery. Methylene chloride (5 mL) was added to the urine sample, and the glass vial was vortex-mixed for 1 minute. The entire contents of the glass vial were transferred to a separatory funnel, and the methylene chloride fraction was collected in a glass test tube. The final methylene chloride extracts were reduced to 200 µL at 40ºC in a Multivap Nitrogen Evaporator (Organomation Associates Inc.) and transferred to amber GC vials with 300 µL inserts for analysis. The samples were then spiked with 10 µL of an internal standard solution containing 20 ng/mL phenanthrene-D10, 20 ng/mL chrysene-D12, and 93 ng/mL of a Carbon Number Distribution Marker (containing n-hexane, n-heptane, n-octane, n-decane, n-dodecane, n-hexadecane, n-heneicosane, n-octacosane, and n-pentatriacontane). During the extraction, plastics were strictly avoided to prevent phthalate contamination. The following were purchased individually from Accustandard: dimethyl phthalate (DMP), diethyl phthalate (DEP), dibutyl phthalate (DBP), di-n-octyl phthalate (DNOP), Bis(2-ethylhexyl) adipate (DEHA), Bis(2-ethylhexyl) phthalate (DEHP), benzyl butyl phthalate (BBP), monobutyl phthalate (MBP), monobenzyl phthalate (MBZP), mono-isobutyl phthalate (MIBP), , monomethyl phthalate (MMP), monoethyl phthalate (MEP), mono(2-ethyl-5-oxohexyl) phthalate (MEOHP), mono(2-ethyl-5-hydroxyhexyl) phthalate (MEHHP), mono(2-ethylhexyl) phthalate (MEHP), mono-isononyl phthalate (MINP), mono(3-carboxypropyl) phthalate (MCPP), monocyclohexyl phthalate (MCHP), and mono-n-octyl phthalate (MOP).

***GC-HRMS for Targeted Analyses of Phthalate Metabolites***

Sample extracts were analyzed using a Thermo GC-Orbitrap QExactive mass spectrometer equipped with a Thermo Trace 1300 GC and TriPlus RSH autosampler. The GC-Orbitrap was a high-resolution mass analyzer, providing up to 120,000 resolving power, 1 ppm mass accuracy, and mass range from 30 to 3,000 m/z to deliver accurate, distinguishable masses for high confidence identification. Data were collected in full-scan mode with resolving power ≥60,000, providing sufficient selectivity and sensitivity. A 3 µl volume of the sample extracts was injected into a 280^o^C split-splitless inlet operated in split-less mode. The sample components were separated on a Restek Rtx-35Sil MS column (30 m x 0.25 mm inner diameter x 0.25 µm film thickness). Helium (99.9999% purity) was used as the carrier gas and was maintained at a flow rate of 1 mL/min. The oven temperature ramp was carried out as follows: 150°C hold for 0.8 min to 200°C at 5°C/min to 300°C at 3°C/min hold 2 min. The transfer line and source temperatures were maintained at 300^o^C and 280^o^C, respectively. Data were collected in full-scan mode with a scan range of 50 to 750 m/z and resolution of 60,000 (ratio of mass to mass difference) between 3 and 45 min.

Targeted analysis was performed using Thermo TraceFinder software (EFS Version 4.1 SP1). The extracted ion chromatogram (XIC) was used for quantification using the most abundant peak in the mass spectrum for each phthalate. Quantification was performed based on an eight-point calibration curve prepared by serial dilution of calibration standards (0.005 to 15 µg/L). The GC-Orbitrap was calibrated prior to each batch run using the certified calibration standards for all expected compounds and internal standards. Compound identity was ensured using retention time and two confirming ions. Limits of detection (LOD) for each compound were determined by injecting 7 samples near the expected limit of detection. Quality Assurance/Quality Control (QA/QC) samples were extracted and analyzed in every sample batch. QC blanks, which were extracted Surine Certified Negative Urine Control samples, were extracted and analyzed every 10 samples and were required to be below the LOD for each compound. Interlaboratory comparisons and proficiency testing were performed using NIST SRM 3074, which contains DMP, DEP, DBP, and BBP, and were used to demonstrate the validity (i.e., precision, accuracy, comparability) of the measurement method. QA/QC check samples, which were negative control urine spiked with a low or high concentration of the phthalate standards, were extracted and analyzed every ten samples to demonstrate repeatability.

**Supplementary Table 2. Targeted Analysis Data for Blanks**

|  | **Detected Concentrations** | | |
| --- | --- | --- | --- |
|  | Mean detected concentration (µg/ L) | Minimum detected concentration (µg/L) | Maximum detected concentration (µg/ L) |
| Mono (2-ethyl-5-hydroxyhexyl) phthalate (MEHHP) | 0.15 | 0.03 | 0.37 |
| Mono (2-ethylhexyl) phthalate (MEHP) | 0.13 | 0.05 | 0.19 |
| Mono (3-carboxypropyl) phthalate (MCCP) | 0.54 | 0.29 | 1.06 |
| Mono-2-ethyl-5-oxohexyl phthalate (MEOHP) | 0.18 | 0.04 | 0.39 |
| Monobenzyl phthalate (MBZP) | 0.11 | 0.03 | 0.25 |
| Monobutyl phthalate (MBP) | 0.68 | 0.49 | 1.02 |
| Monocyclohexyl phthalate (MCHP) | 0.12 | 0.06 | 0.24 |
| Monoethyl phthalate (MEP) | <LOD | <LOD | <LOD |
| Monoisobutyl phthalate (MIBP) | 0.68 | 0.49 | 1.02 |
| Monoisononyl phthalate (MINP) | 0.01 | 0.01 | 0.01 |
| Monomethyl phthalate (MMP) | <LOD | <LOD | <LOD |
| Mono-n-octyl phthalate (MOP) | 0.05 | 0.03 | 0.09 |
| Benzyl butyl phthalate (BBP) | 0.15 | 0.03 | 0.37 |
| Bis(2-ethylhexyl) adipate (DEHA) | 0.12 | 0.03 | 0.19 |
| Bis(2-ethylhexyl) phthalate (DEHP) | 0.24 | 0.06 | 0.47 |
| Dibutyl phthalate (DBP) | 0.18 | 0.04 | 0.39 |
| Diethyl Phthalate (DEP) | 0.11 | 0.03 | 0.25 |
| Dimethyl phthalate (DMP) | 0.04 | 0.02 | 0.07 |
| Di-n-octyl phthalate (DNOP) | 0.12 | 0.06 | 0.24 |

Recoveries were consistent within batches of samples.

**Supplementary Table 3. Targeted Analysis Data for Matrix Spikes and Recovery**

|  |  | **Detected Concentrations** | | | **Percent Recovery** | | |
| --- | --- | --- | --- | --- | --- | --- | --- |
|  | Expected concentration (µg/ L) | Mean detected concentration (µg/ L) | Minimum detected concentration (µg/ L) | Maximum detected concentration (µg/ L) | Average Recovery (n=5) | Standard Deviation (n=5) | Confidence Interval (α=0.05) (n=5) |
| Mono (2-ethyl-5-hydroxyhexyl) phthalate (MEHHP) | 2.50 | 2.96 | 2.38 | 3.74 | 118.5% | 21.6% | 26.8% |
| Mono (2-ethylhexyl) phthalate (MEHP) | 2.50 | 3.27 | 2.81 | 3.69 | 130.6% | 16.9% | 20.9% |
| Mono (3-carboxypropyl) phthalate (MCCP) | 2.50 | 1.94 | 1.19 | 2.88 | 77.8% | 30.6% | 38.0% |
| Mono-2-ethyl-5-oxohexyl phthalate (MEOHP) | 2.50 | 3.19 | 2.78 | 3.86 | 127.6% | 17.0% | 21.1% |
| Monobenzyl phthalate (MBzP) | 2.50 | 2.82 | 1.86 | 4.12 | 112.9% | 34.0% | 42.3% |
| Monobutyl phthalate (MBP) | 2.50 | 3.07 | 2.28 | 4.41 | 122.6% | 32.0% | 39.7% |
| Monocyclohexyl phthalate (MCHP) | 2.50 | 2.28 | 1.93 | 3.06 | 91.2% | 18.8% | 23.4% |
| Monoethyl phthalate (MEP) | 2.50 | 2.74 | 2.09 | 3.77 | 109.5% | 25.6% | 31.8% |
| Monoisobutyl phthalate (MIBP) | 2.50 | 3.07 | 2.28 | 3.85 | 122.6% | 29.6% | 36.7% |
| Monoisononyl phthalate (MINP) | 2.50 | 3.12 | 2.52 | 3.55 | 124.6% | 16.2% | 20.1% |
| Monomethyl phthalate (MMP) | 2.50 | 2.57 | 2.23 | 2.83 | 102.7% | 10.8% | 13.4% |
| Mono-n-octyl phthalate (MOP) | 2.50 | 3.61 | 2.79 | 4.29 | 144.3% | 27.8% | 34.5% |
| Benzyl butyl phthalate (BBP) | 2.50 | 2.80 | 2.12 | 3.82 | 112.2% | 28.8% | 35.7% |
| Bis(2-ethylhexyl) adipate (DEHA) | 2.50 | 2.88 | 1.04 | 4.09 | 115.0% | 47.4% | 58.9% |
| Bis(2-ethylhexyl) phthalate (DEHP) | 2.50 | 2.83 | 2.20 | 3.58 | 113.4% | 21.1% | 26.2% |
| Dibutyl phthalate (DB)) | 2.50 | 2.59 | 2.24 | 2.90 | 103.4% | 12.3% | 15.2% |
| Diethyl Phthalate DEP) | 2.50 | 2.75 | 2.18 | 3.41 | 110.1% | 23.2% | 28.8% |
| Dimethyl phthalate (DMP) | 2.50 | 3.04 | 2.25 | 5.10 | 121.5% | 47.1% | 58.5% |
| Di-n-octyl phthalate (DNOP) | 2.50 | 2.55 | 2.07 | 2.76 | 102.0% | 11.0% | 13.6% |

**Supplemental Table 4. Sociodemographic Data of Participants Who Provided a Urine Sample Compared to Those Who Did Not**

| Participant characteristics | Urine  (N=21) | No Urine  (N=25) | P-value |
| --- | --- | --- | --- |
| Age, y (range) | 33.10 ± 4.81  (22-41) | 27.16 ± 5.22  (19-37) | **0.0003** |
| Race, n (%) |  |  |  |
| Black Hispanic | 5 (23.8) | 8 (32.0) | 0.43 |
| Black non-Hispanic | 5 (23.8) | 3 (12.0) |  |
| AI/AN Hispanic | 1 (4.8) | 0 |  |
| White Hispanic | 0 | 3 (12.0) |  |
| Greek Hispanic | 1 (4.8) | 0 |  |
| Taino Hispanic | 1 (4.8) | 0 |  |
| Asian non-Hispanic | 0 | 1 (4.0) |  |
| Black and AI/AN | 1 (4.8) | 0 |  |
| Other Hispanic | 6 (28.6) | 8 (32.0) |  |
| Refuse to report and Hispanic | 1 (4.8) | 2 (8.0) |  |
| Country, n (%) |  |  |  |
| Dominican Republic | 9 (42.9) | 11 (44.0) | 0.84 |
| United States | 10 (47.6) | 12 (48.0) |  |
| Guinea | 1 (4.8) | 0 |  |
| Togo | 0 | 1 (4.0) |  |
| Liberia | 1 (4.8) | 0 |  |
| St. Thomas | 0 | 1 (4.0) |  |
| Language, n (%) |  |  |  |
| English | 20 (95.2) | 21 (84.0) | 0.36 |
| Spanish | 1 (4.8) | 4 (16.0) |  |
| Pregnancy, wk | 31.48 ± 3.39  (25-37) | 31.28 ± 3.29  (22-36) | 0.84 |

| **BASELINE** | **GM** | **GSD** | **MIN** | **25th** | **50th** | **75th** | **MAX** |
| --- | --- | --- | --- | --- | --- | --- | --- |
| **DBP** | 3.06 | 1.83 | 0.51 | 2.57 | 3.29 | 4.69 | 5.82 |
| **DEP** | 0.21 | 4.72 | 0.003 | 0.15 | 0.33 | 0.52 | 1.37 |
| **DMP** | 0.38 | 2.07 | 0.06 | 0.35 | 0.42 | 0.61 | 0.80 |
| **MBP** | 17.33 | 2.26 | 2.68 | 13.09 | 17.97 | 27.65 | 77.83 |
| **MEP** | 2.20 | 49.08 | 0.07 | 0.07 | 0.07 | 131.10 | 202.42 |
| **MIBP** | 7.85 | 2.92 | 0.79 | 5.36 | 9.66 | 15.46 | 38.75 |
| **FOLLOW-UP 1** |  |  |  |  |  |  |  |
| **DBP** | 2.11 | 2.22 | 0.54 | 1.15 | 2.44 | 3.53 | 9.51 |
| **DEP** | 0.26 | 3.12 | 0.02 | 0.14 | 0.29 | 0.62 | 1.23 |
| **DMP** | 0.59 | 1.46 | 0.17 | 0.56 | 0.63 | 0.72 | 0.82 |
| **MBP** | 18.47 | 3.75 | 3.19 | 8.75 | 10.59 | 31.95 | 424.25 |
| **MEP** | 2.52 | 57.84 | 0.07 | 0.07 | 0.07 | 174.68 | 368.34 |
| **MIBP** | 9.33 | 5.94 | 0.24 | 3.15 | 8.65 | 27.10 | 324.37 |

**Supplementary Table 5. Phthalates and Phthalate Esters Eligible for Analysis Within the Let’s ROAR Pilot Study: Limit of Detection and Percentage of Non-Detect**

| **Parent Compound** | **% Non-Detect** | **LOD (** **µg/ L)** | **Urinary Metabolite** | **% Non-Detect** | **LOD (** **µg/ L)** |
| --- | --- | --- | --- | --- | --- |
| Di-butyl phthalate (DBP) | 0 | 0.066 | Monobutyl phthalate (MBP) | 0 | 0.063 |
| Di-ethyl phthalate (DEP) | 0 | 0.010 | Monoethyl phthalate (MEP) | 65 | 0.094 |
| Di-methyl phthalate (DMP) | 0 | 0.034 |  |  |  |
|  |  |  | Monoisobutyl phthalate (MIBP) | 0 | 0.050 |

**“Eligible for analysis” was defined by participants with baseline and follow up data.**

**Supplemental Table 6. Phthalates and Phthalate Esters Descriptive Statistics**
